# Prediction of Mutations to Control Pathways Enabling Tumor Cell Invasion with the CoLoMoTo Interactive Notebook (Tutorial)

**DOI:** 10.3389/fphys.2018.00787

**Published:** 2018-07-06

**Authors:** Nicolas Levy, Aurélien Naldi, Céline Hernandez, Gautier Stoll, Denis Thieffry, Andrei Zinovyev, Laurence Calzone, Loïc Paulevé

**Affiliations:** ^1^LRI UMR 8623, Centre National de la Recherche Scientifique, Université Paris-Sud, Université Paris-Saclay, Orsay, France; ^2^École Normale Supérieure de Lyon, Lyon, France; ^3^Computational Systems Biology Team, Institut de Biologie de l'École Normale Supérieure, Centre National de la Recherche Scientifique UMR8197, INSERM U1024, École Normale Supérieure, PSL Université, Paris, France; ^4^Université Paris Descartes, Sorbonne Paris Cité, Paris, France; ^5^Équipe 11 Labellisée Ligue Nationale contre le Cancer, Centre de Recherche des Cordeliers, Paris, France; ^6^Institut National de la Santé et de la Recherche Médicale, Paris, France; ^7^Université Pierre et Marie Curie, Paris, France; ^8^Metabolomics and Cell Biology Platforms, Gustave Roussy Cancer Campus, Villejuif, France; ^9^Institut Curie, PSL Research University, Paris, France; ^10^INSERM U900, Paris, France; ^11^MINES ParisTech, PSL Research University, CBIO-Centre for Computational Biology, Paris, France; ^12^Lobachevsky University, Nizhni Novgorod, Russia

**Keywords:** Boolean networks, stochastic simulations, model verification, software tools, reproducibility

## Abstract

Boolean and multi-valued logical formalisms are increasingly used to model complex cellular networks. To ease the development and analysis of logical models, a series of software tools have been proposed, often with specific assets. However, combining these tools typically implies a series of cumbersome software installation and model conversion steps. In this respect, the *CoLoMoTo Interactive Notebook* provides a joint distribution of several logical modeling software tools, along with an interactive web Python interface easing the chaining of complementary analyses. Our computational workflow combines (1) the importation of a GINsim model and its display, (2) its format conversion using the Java library BioLQM, (3) the formal prediction of mutations using the OCaml software Pint, (4) the model checking using the C++ software NuSMV, (5) quantitative stochastic simulations using the C++ software MaBoSS, and (6) the visualization of results using the Python library matplotlib. To illustrate our approach, we use a recent Boolean model of the signaling network controlling tumor cell invasion and migration. Our model analysis culminates with the prediction of sets of mutations presumably involved in a metastatic phenotype.

## 1. Introduction

Boolean and multi-valued logical formalisms are increasingly used to model complex cellular networks (see e.g., Helikar et al., 2012; Zaudo and Albert, 2015; Collombet et al., 2017). A logical model is usually defined in three steps:

1. The delineation of a regulatory graph, where the vertices (nodes) represent signaling or regulatory components (proteins, genes, microRNAs, etc.), while the arcs (arrows) represent regulatory interactions between pairs of components. These arcs are labeled by a sign: positive in the case of activation, negative in the case of an inhibition (multiple arcs between two nodes may be considered but are not used here).
2. A discrete variable is associated with each node. In the simplest cases, as hereafter, these variables are Boolean, i.e., they can take only two values (0 or 1), denoting the absence/inactivity or the presence/activity of the corresponding components.
3. Finally, a logical rule is associated with each component to specify the combinations enabling its activation. More precisely, this rule combines the different variables corresponding to the regulatory components using the logical negation (denoted by !), conjunction (denoted by &) and disjunction (denoted by |). For example, the rule associated with the component GF in the model considered below is !CDH1 & (GF | CDH2), which reads as “the component GF will be activated in the absence of CDH1 and in the presence of CDH2 or GF itself.” In other words, CDH2 is required transiently for GF activation, in the absence of CDH1.

To support the development and analysis of logical models, a series of software tools have been proposed, often with specific assets (Naldi et al., 2009; Klarner et al., 2017; Paulevé, 2017; Stoll et al., 2017).

The *CoLoMoTo Interactive Notebook*^1^ (Naldi et al., 2018b) relies on Docker^2^ and Jupyter^3^ technologies to assist on editing and sharing reproducible analysis workflows for logical models. In addition to the distribution of a set of software tools to define and analyse Boolean and multi-valued networks, a unified Python interface for each of the integrated tools is provided, greatly easing the execution and chaining of complementary analyses.

This protocol describes in details the usage of the CoLoMoTso Interactive Notebook to provide a reproducible analysis of a recently published model of the signaling network controlling tumor cell invasion and migration. More specifically, we combine different tools (Table 1) to compute the model stable states, perform stochastic simulations, compute (sets of) mutations controlling the reachability of specific stable states, and evaluate their efficiency.

**Table 1:** List of software tools used in this notebook.

**Tool**	**Website**	**Role in this notebook**
GINsim	ginsim.org	Model input and display, conversion to bioLQM and NuSMV
bioLQM	colomoto.org/biolqm	Fixpoint computation, conversion to MaBoSS and Pint
MaBoSS	maboss.curie.fr	Stochastic simulations, assess impact of mutations on propensity of reaching phenotypes
Pint	loicpauleve.name/pint	Formal prediction of mutants
NuSMV	nusmv.fbk.eu	Formal verification of phenotypes reachability and stability

## 2. Materials and equipment

### 2.1. Executable and reproducible model analysis

This protocol has been actually edited entirely as a Jupyter notebook before being converted to a LaTeX document for journal-specific editing purposes. The original notebook file is provided as Supplemental Material. It can also be visualized and downloaded for execution in the CoLoMoTo Interactive Notebook at https://nbviewer.jupyter.org/gist/pauleve/a86717b0ae8750440dd589f778db428f/Usecase%20-%20Mutations%20enabling%20tumour%20invasion.ipynb.

The blocks beginning with In [.] correspond to Jupyter *code cells*, which contain the Python instructions to execute. When relevant, the blocks beginning with Out [.] display the result of the last instruction of the corresponding code cell.

Provided Docker and Python are installed, the CoLoMoTo Interactive notebook can be installed by typing and executing the following command^4^ on GNU/Linux, macOS, and Microsoft Windows:


pip install -U colomoto-docker


Once installed, the notebook can be executed by typing


colomoto-docker -V 2018-05-29


The execution of this command will open a web page with the Jupyter notebook interface, enabling the loading and execution of the code. Note that “SHIFT+ENTER” must be used to execute each code cell. More information on colomoto-docker usage can be obtained by typing colomoto-docker --help and by visiting https://github.com/colomoto/colomoto-docker.

### 2.2. Notebook preparation

This notebook makes use of the following Python modules:


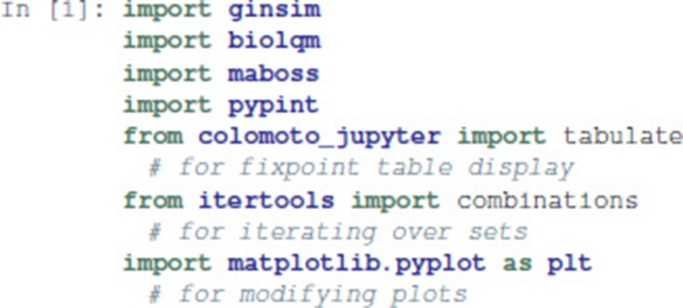


## 3. Stepwise procedures

### 3.1. Model

We analyse a Boolean model of the signaling network controlling cell tumor invasion, which was recently reported in Cohen et al. (2015). This model can be loaded directly from the GINsim model repository at http://ginsim.org/models_repository.

We first show how to use GINsim (Naldi et al., 2018a) to fetch and parse the GINML file (GINsim graph-based XML format, encapsulated in a zginml archive) and display the regulatory graph of the network. To load the model, we copied the URL of the .zginml file from the model repository page at http://ginsim.org/node/191. The file is also available as Supplemental Data (Data Sheet 1).


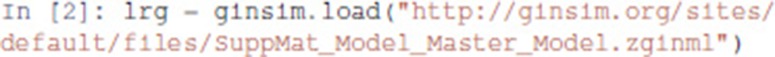


The regulatory graph (using the graphical setting specified in the model file) can be displayed with the following command:


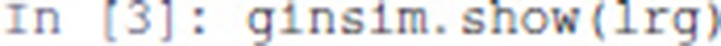


**Figure 1:**
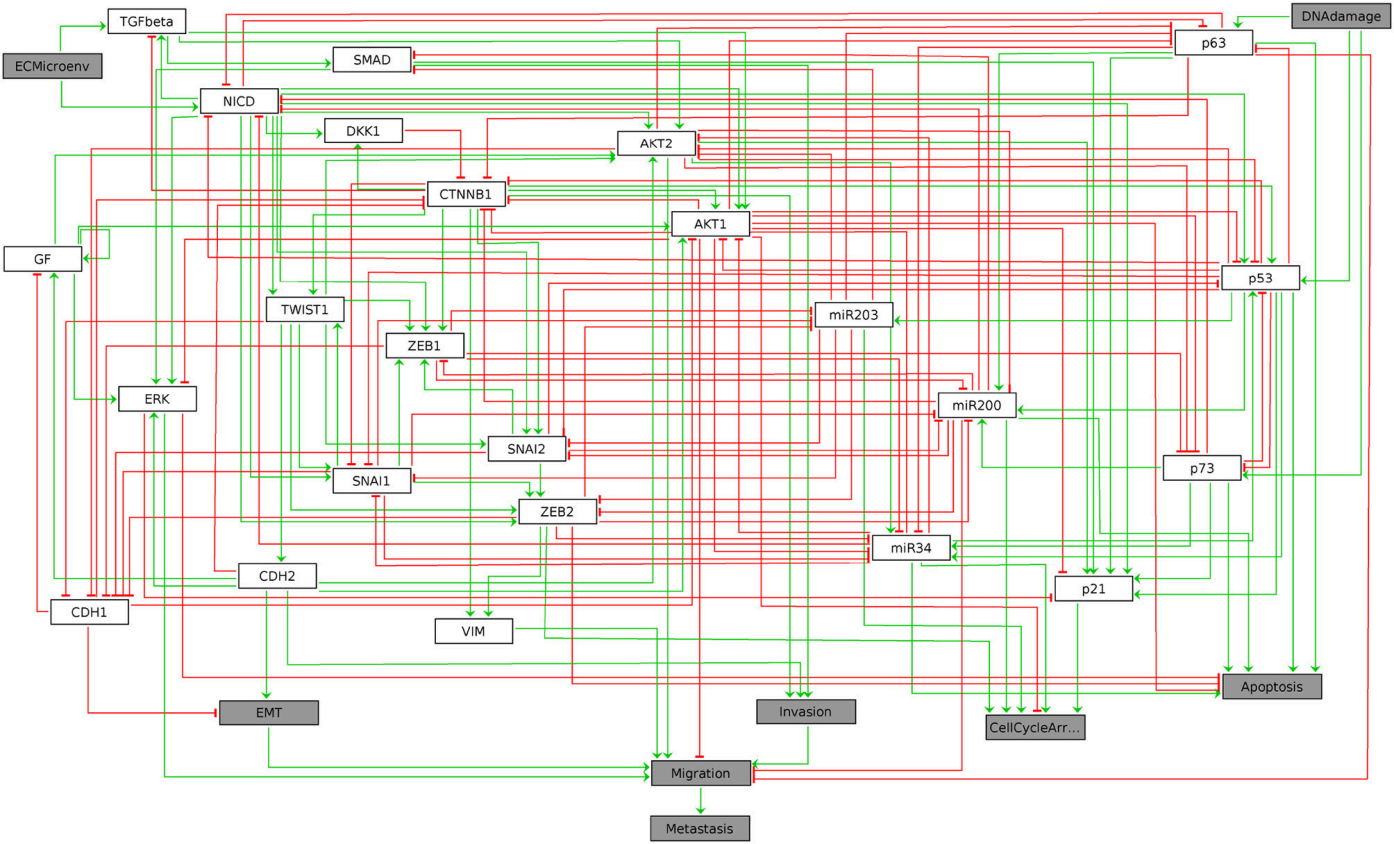
Graphical output resulting from the input code: In [3]: ginsim.show(lrg).

The resulting graphics is reproduced in Figure 1.

In this regulatory graph, the gray boxes denote input and output vertices (nodes). Green arrows and red T arrows respectively denote activatory and inhibitory interactions. A set of rules combining the vertices with the Boolean operators NOT, AND, and OR, which must be consistent with the regulatory graph, then allows the computation of enabled transitions for each network state. These rules have been defined in Cohen et al. (2015) and are specified within the GINsim model.

### 3.2. Identification of stable states

First, we compute the complete list of logical stable states (or fixpoints) of the model using the Java library bioLQM (Naldi, 2018). We thus need to convert the GINsim model into bioLQM:


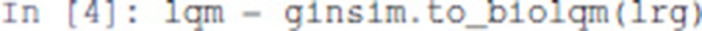


At that stage, lrg is a Python object representing the model suitable for GINsim, and lqm is a Python object representing the equivalent model suitable for bioLQM.

The list of stable states of a bioLQM model is computed as follows:


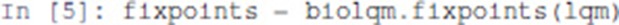


Here, fixpoints is a Python list of states. A state is encoded as a Python association table (dictionary), which maps each node of the network to a value.

For a nice display of the list of stable states, one can use the tabulate function provided in the colomoto_jupyter Python library, imported at the beginning of the notebook:


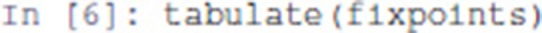


**Figure 2:**
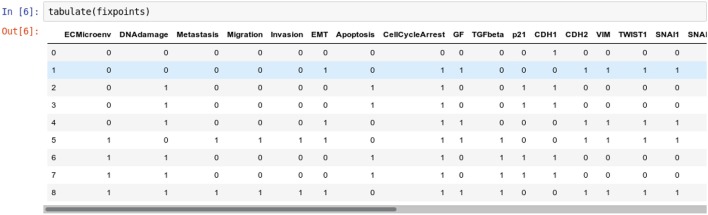
Graphical output resulting from the input code: In [6]: tabulate(fixpoints).

Figure 2 shows the table as displayed in the notebook. The complete table is given in Supplemental Data.

It results that the model has nine stable states, each corresponding to a row in the table, four of which enable apoptosis (rows with value 1 in fourth column “Apoptosis”). Note that the input node DNAdamage is also active in each of these four states.

A state can be visualized on the regulatory graph using GINsim. For example, the third stable state can be displayed using the following command:


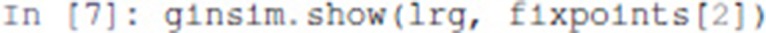


**Figure 3:**
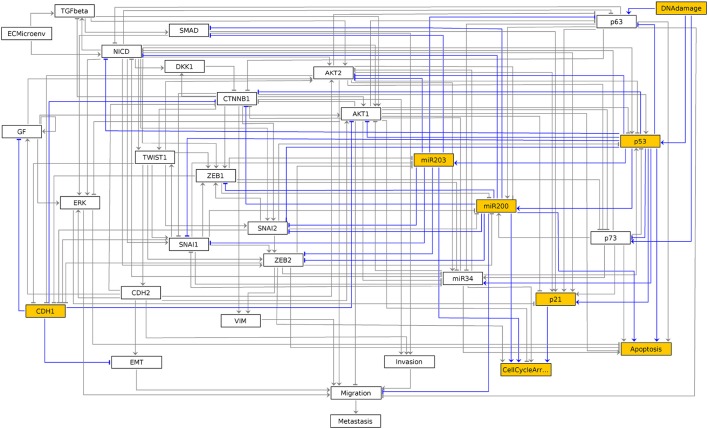
Graphical output resulting from the input code: In [7]: ginsim.show(lrg, fixpoints[2]).

The resulting graphics is reproduced in Figure 3.

In this graph, the vertices shown in white or orange denote components that are OFF (value 0) or ON (value 1) respectively.

### 3.3. Assessing the probabilities to reach alternative attractors using MaBoSS

MaBoSS (Stoll et al., 2017) is a C++ software enabling the stochastic simulation of Boolean networks by translating them into continuous time Markov processes. Each node activation and inactivation is associated with an *up* and a *down rate*, which specify the propensity of the corresponding transitions. From a given state, the simulation integrates all the possible node updates and derives a probability and a duration for each transition. By default, all transitions are assigned the same rate. For a given set of initial conditions, MaBoSS produces time trajectories and estimates probabilities of model states over the whole simulation time. Steady state distributions can thus be approximated, provided that a sufficient number of sufficiently long simulations have been performed.

The aim of this section is to reproduce part of the results obtained by Cohen et al. (2015), which show that a Notch (NICD) gain-of-function together with a p53 loss-of-function prevent reaching a stable apoptotic phenotype.

First, we convert the bioLQM model to MaBoSS:


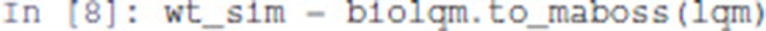


The variable wt_sim is a Python object that gathers both the Boolean network rules and the settings for the simulations, including the transition rates.

#### 3.3.1. Simulation setup

The stochastic simulation of Boolean networks with MaBoSS requires the specification of several parameters.

##### 3.3.1.1. Initial states

First, a distribution of initial states must be specified: each simulation then starts from a state sampled from this distribution. The distribution is determined by assigning a probability to start in state 0 or in state 1 to each node. By default, a node has a probability 1 to start in state 0.

The maboss Python library provides *widgets* to ease the assignment of this initial distribution. The following code enables the definition of a distribution of initial states with all nodes at 0, except DNAdamage and ECMicroenv with equiprobable 0 and 1 values. After pressing “OK,” the notebook cell will be replaced by the actual Python call resulting in equal probabilities for these two nodes to start in active or inactive states.


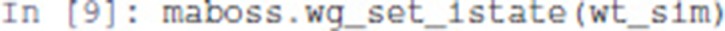


**Figure 4:**
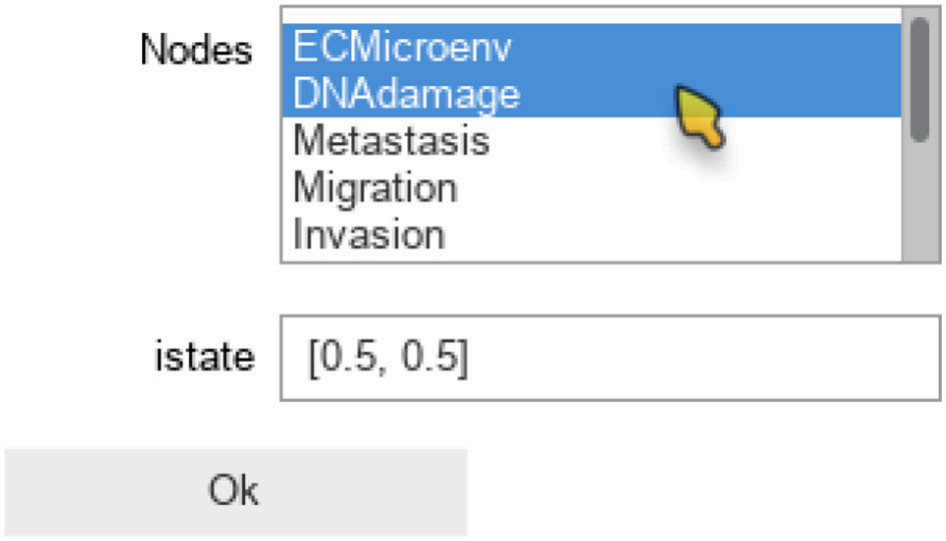
Graphical output resulting from the input code: In [9]: maboss.wg_set_istate(wt_sim).

The notebook will then display the widgets reproduced in Figure 4. The selection of nodes and of initial conditions shown in this figure are then translated in the following code:


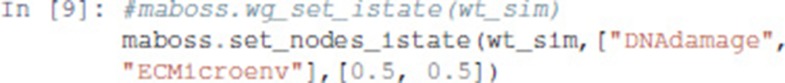


##### 3.3.1.2. Output nodes

Using MaBoSS, we can focus on the *output* nodes and ignore the other nodes, which enable us to identify the corresponding phenotypes. This can be done using the following code:


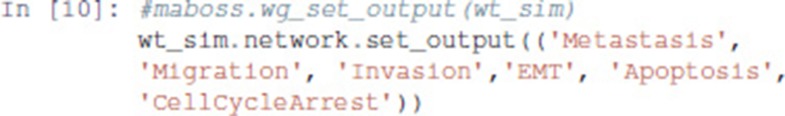


##### 3.3.1.3. Simulation parameters

The update_parameters method can be used to specify several parameters for the stochastic simulation algorithm. We show below the complete list of parameters with the values obtained by default when translating a model from GINsim. The method can be called with any subset of these parameters.

Among the parameter list, sample_count corresponds to the number of simulations performed to compute statistics, while max_time is the maximum (simulated) duration of a trajectory. Note that for a proper estimation of probabilities of the stable states, max_time needs to be long enough for the simulation to reach an asymptotic solution.


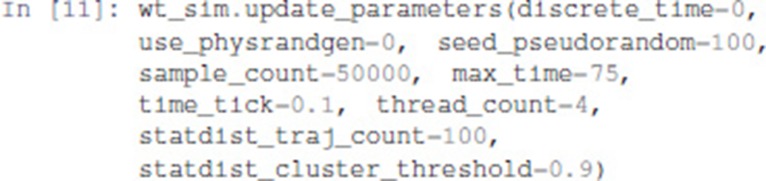


#### 3.3.2. Simulation of the wild-type model

The object wt_sim represents the input of MaBoSS, encompassing both the network and simulation parameters. The simulations are triggered with the .run() method and return a Python object for accessing the results.


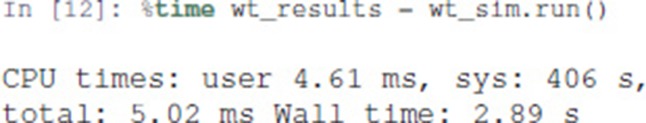


The resulting object gives access to the output data generated by MaBoSS. It includes notably the mean probability over time for the activity of the output states integrated over all the performed simulations.

The function plot_piechart displays proportionally the mean probability of each output state at the *last* time point. Provided the simulation time has been set high enough, this gives an approximation of the probabilities of the stable states reachable from the specified initial conditions.


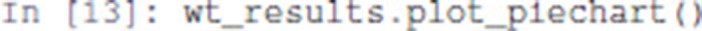


**Figure 5:**
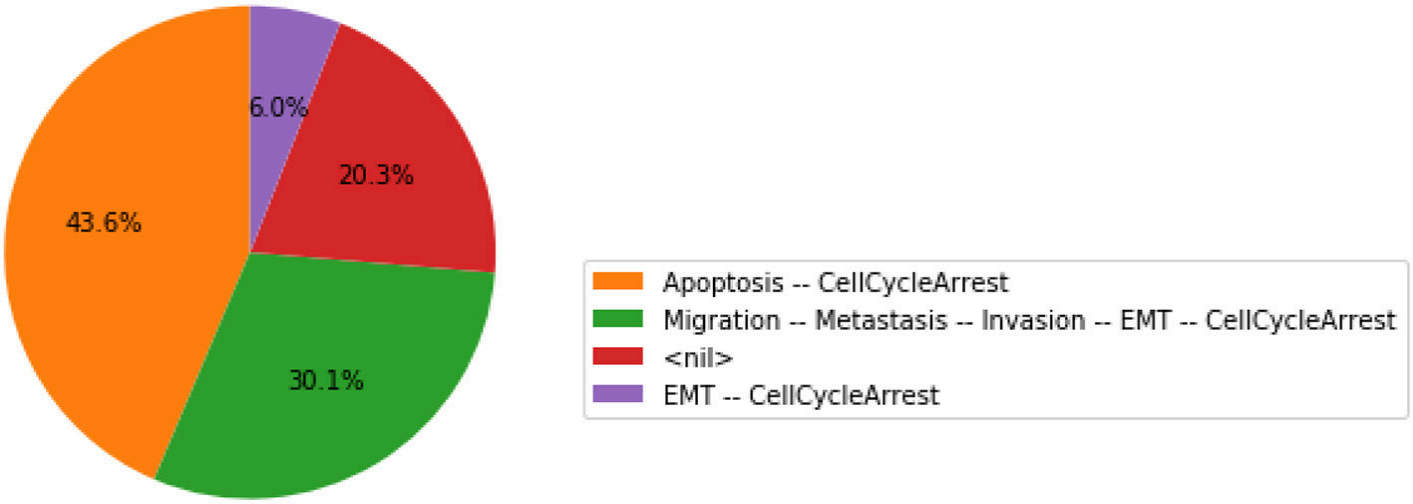
Graphical output resulting from the input code: In [13]: wt_results.plot_piechart().

The resulting graphics is reproduced in Figure 5.

In this chart, a state is described by the set of its active output nodes and is associated to a phenotype. For instance, the “<nil>” phenotype has all output nodes set to 0, which was referred to as the “homeostatic state” in the original article; in the case of the “Apoptosis -- CellCycleArrest” phenotype, the two output nodes Apoptosis and CellCycleArrest are simultaneously active, while the other output nodes are inactive; the “EMT -- CellCycleArrest” phenotype denotes cells that have gone through the epithelial to mesenchymal transition (EMT), but did not invade the tissue, hence the output nodes Invasion, Migration and Metastasis are inactive; finally the “Migration -- Metastasis -- Invasion -- EMT -- CellCycleArrest” phenotype corresponds to a metastatic state, i.e., to cells that went through EMT, invaded the tissue and migrated to a distant site.

From this plot, we can deduce that, from the specified set of initial conditions, the apoptotic state (orange section), the EMT (purple section) and the metastatic states (green section) can be reached (the proportion of simulations that reached none of these phenotypes correspond to the red section).

The mean value of each output node during the simulations can be plotted with the following command:


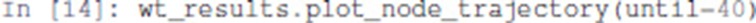


**Figure 6:**
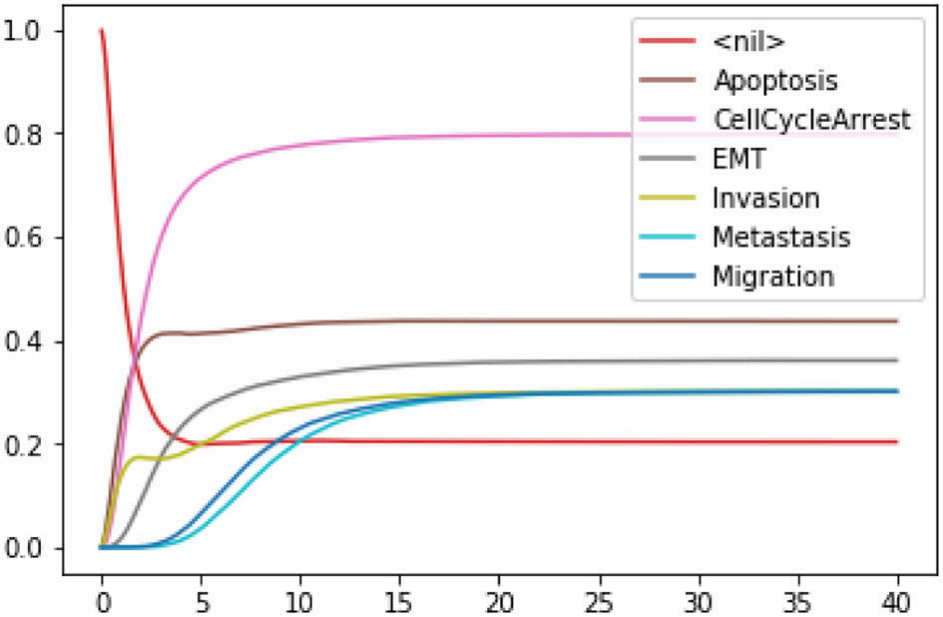
Graphical output resulting from the input code: In [14]: wt_results.plot_node_trajectory(until=40).

The resulting graphics is reproduced in Figure 6.

#### 3.3.3. Simulation of double mutant notch++/p53--

In the original article (Cohen et al., 2015), the authors analyzed the double Notch++/p53-- mutant, i.e., the combination of a Notch gain-of-function combined with a p53 loss-of-function, showing that all trajectories lead to a metastatic state.

A mutant can be configured by copying the wild-type model, and use the mutate method to model the desired gains and losses of function:


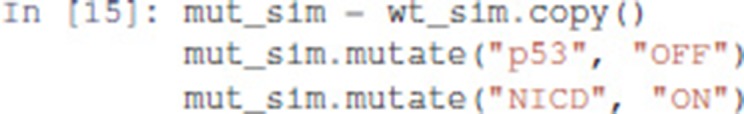


The modified model can then be simulated exactly as for the wild-type case:


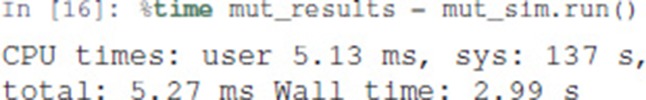



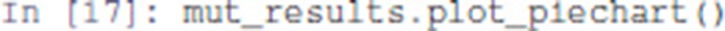


**Figure 7:**
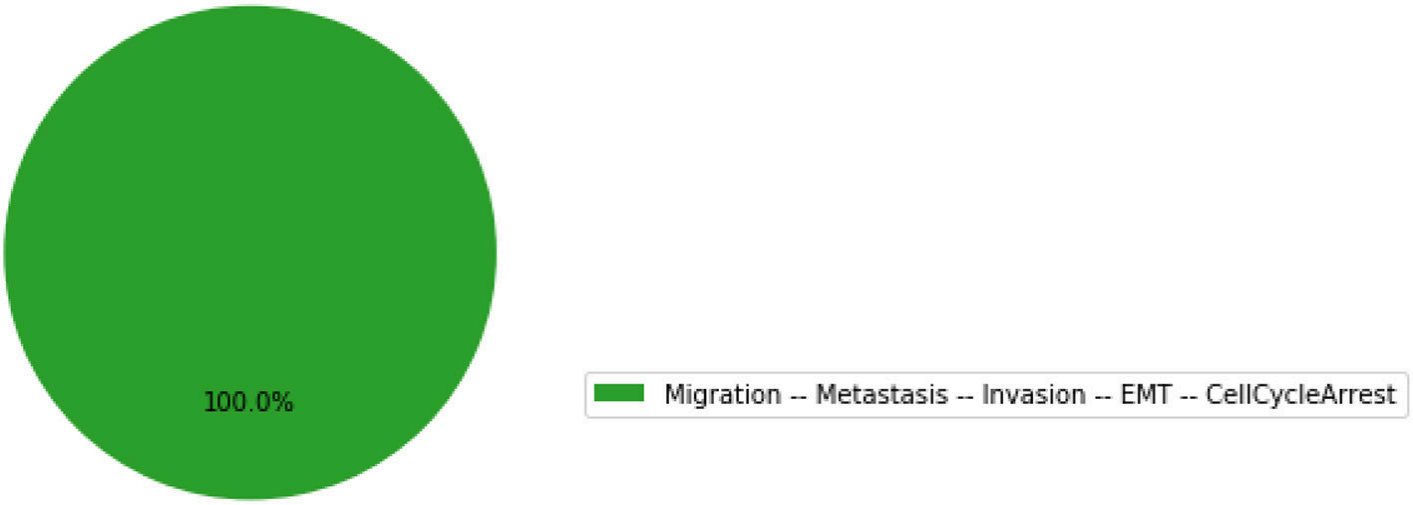
Graphical output resulting from the input code: In [17]: mut_results.plot_piechart().

The resulting graphics is reproduced in Figure 7.

Using the same parameters as for the wild-type model, all the trajectories obtained for the double mutant model reach the metastatic invasive state exclusively. This suggests that such a double mutation can be responsible for a loss of apoptotic capability of cancer cells.

### 3.4. Formal analysis with pint and NuSMV

In the above section, the conclusion regarding the loss of apoptotic stable state relies on stochastic simulations, which, in general, may not offer a complete coverage of the possible trajectories. Therefore, one may want to formally verify whether the loss of reachable stable apoptosis state is total or not. First, we show how to use Pint (Paulevé, 2017) to predict combinations of mutations which are guaranteed to prevent the activation of apoptosis. Next, we use the software NuSMV (Cimatti et al., 2002) to evaluate formally the Notch++/p53-- double mutant. Finally, we use MaBoSS to assess the efficiency of new combinations of mutations predicted by Pint.

#### 3.4.1. Formal predictions of mutations from the wild-type model

Pint implements formal methods that allow deducing combinations of mutations guaranteed to block the reachability of a given state.

First, we convert the bioLQM model to Pint:


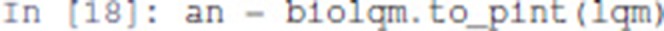


Then, we transfer the initial conditions defined in MaBoSS to the Pint model an. Like MaBoSS, Pint supports multiple initial values for a single node. However, in contrast to MaBoSS, Pint does not consider probability distributions.


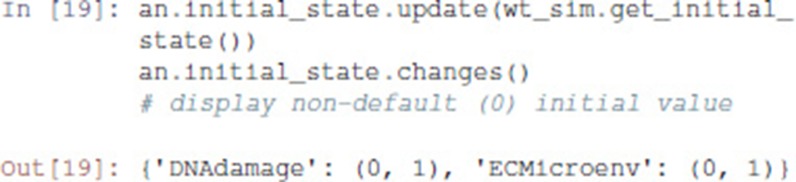


Given a (partial) state specification, Pint provides the method oneshot_mutations_for_cut, which returns different sets of mutations guaranteed to prevent any trajectory from any possible initial state to reach, *even transiently*, the specified state.


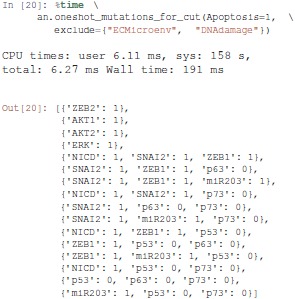


Among the returned mutation sets, one can spot the mutation {'NICD': 1, 'p53': 0, 'p73': 0}, which combines a gain-of-function of Notch ('NICD': 1) with a loss-of-function of p53 ('p53': 0), along with a loss-of-function of p73 ('p73': 0).

Noteworthy, forbidding *transient* reachability entails a stronger constraint than just preventing any *stable* state with the specified property. Indeed, some mutations may remove the stability of the specified states, while some trajectories may still traverse these states, but only transiently.

Therefore, the sets of mutations returned by Pint, albeit correct, might be non-minimal for controlling only the long-term dynamics of the system. Finally, note that the analysis of Pint can give incomplete results. This is due to the technology on which the computation relies (static analysis), which allows addressing very large scale networks.

#### 3.4.2. Revisiting the notch++/p53-- double mutant

We will first formally analyse the Notch++/p53-- double mutant to show that asymptotic apoptosis is forbidden, although transient activation of apoptosis node might still be possible.

One can apply a mutation on a Pint model using the lock method. A new model is returned with a constant value for the corresponding nodes.


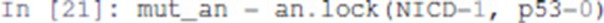


Then, we use the temporal logic CTL (Clarke and Emerson, 1982) to specify formally the dynamical properties to verify. CTL expression can be built using the colomoto.temporal_logics Python module.


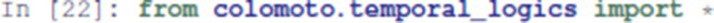


First, the existence of a trajectory leading to a *transient* state where Apoptosis is active can be specified as follows:


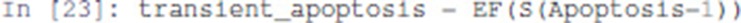


EF is a temporal logic operator that is true if there exists at least one trajectory leading to a state verifying the properties given as argument. Here the property S(Apoptosis=1) specifies that the state has the node Apoptosis active.

Next, the existence of a trajectory leading to a *stable* Apoptosis activation can be specified as follows:


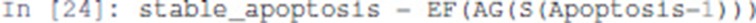


Here, AG enforces that *all* the states reachable via any trajectory have the node Apoptosis active.

Finally, we gather these two properties in a Python dictionary for later use:


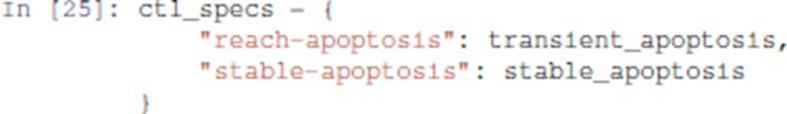


The adequation of a model with a CTL property can be assessed using a *model-checker* such as NuSMV (Abou-Jaoudé et al., 2015).

Pint provides a conversion to NuSMV models. By default, the NuSMV model considers any initial state. With the skip_init=False option, we enforce that the properties are verified only from the initial states defined earlier.


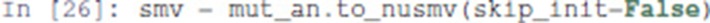


We then add the properties defined above, and ask NuSMV to verify them.


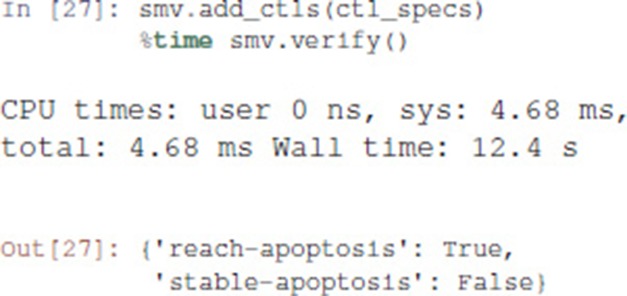


Interestingly, the Notch++/p53-- double mutant can still reach an apoptotic state, but only transiently: the property stable-apoptosis being false, it is guaranteed that all trajectories eventually lead to stable apoptosis inactivation.

To complete our analysis, we now consider the triple mutant obtained by adding a loss-of-function of p73. As predicted by Pint, transient reachability of apoptosis is impossible in this triple mutant. We can use NuSMV to further verify that it is the case, using the following code:


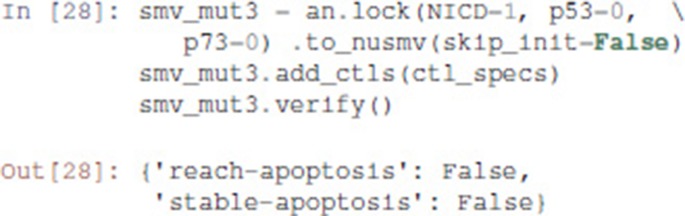


#### 3.4.3. Analysis of formally predicted SNAI2++/ZEB1++/miR203++ triple mutant

The mutant combinations predicted with Pint should be refined when the aim is to control specifically stable behaviors. In general, given a set of mutations guaranteed to block any transient activation of a node, one may verify whether only a subset of them are sufficient to achieve proper control of the sole stable states.

We show here how we can take advantage of the Python environment to provide a small program, which, for each subset of mutations of a multiple mutant (here a triple gain-of-function for SNAI2, ZEB1 and miR203), performs stochastic simulations with MaBoSS to assess the probabilities to reach the different stable behaviors from the specified set of states.

The computation can take a couple of minutes. The results are shown in a graphical form (colored pie charts) for each single and double loss-of-function combination. In the pie charts, “Others” regroup states with an individual probability less than 1%, which often correspond to simulated trajectories having not reached an attractor in the given amount of time.


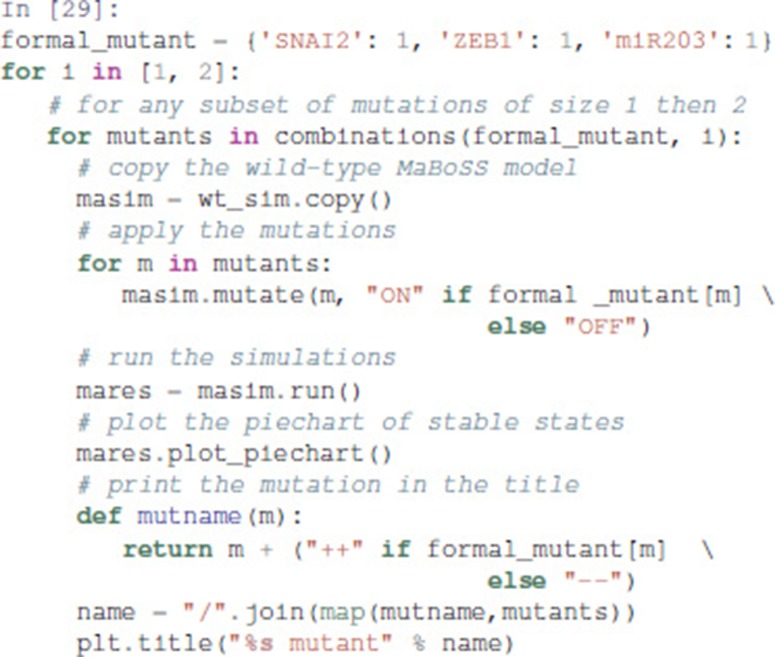


**Figure 8:**
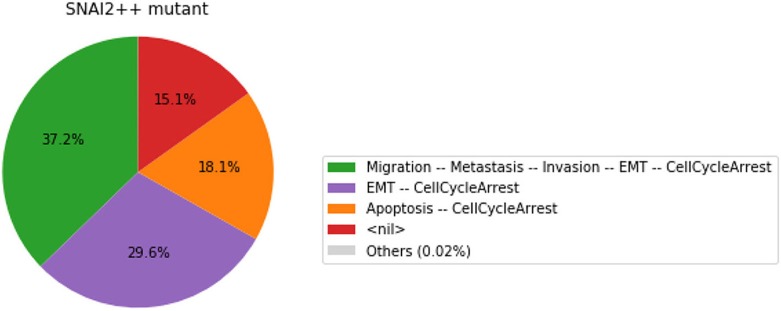
Graphical output resulting from the input code: In [29].

**Figure 9:**
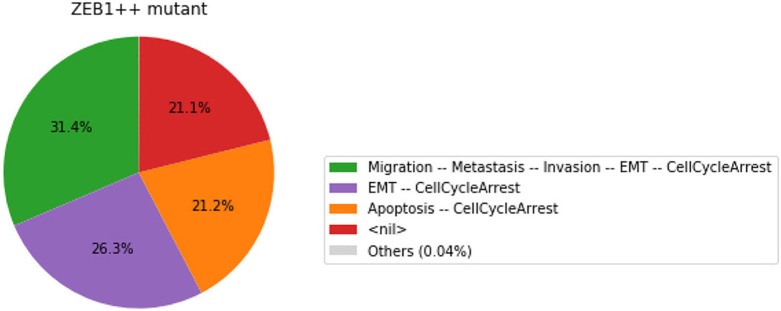
Graphical output resulting from the input code: In [29].

**Figure 10:**
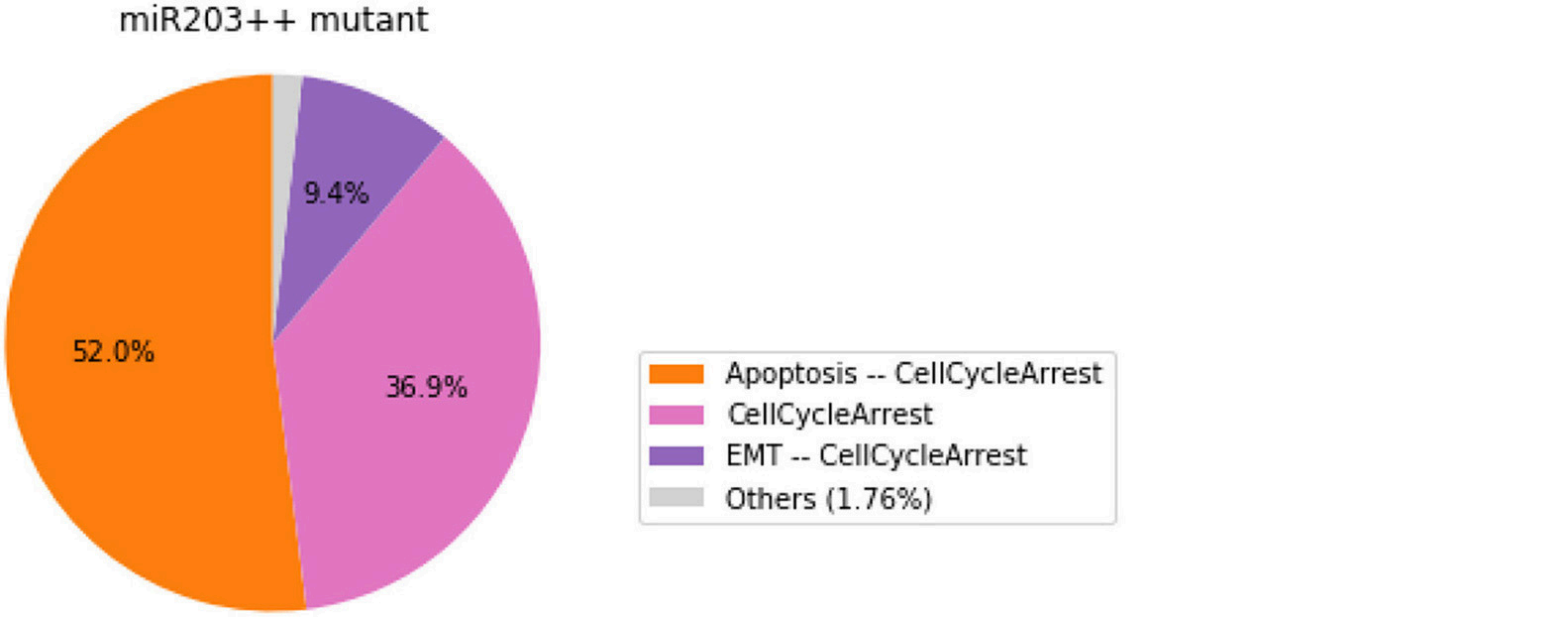
Graphical output resulting from the input code: In [29].

**Figure 11:**
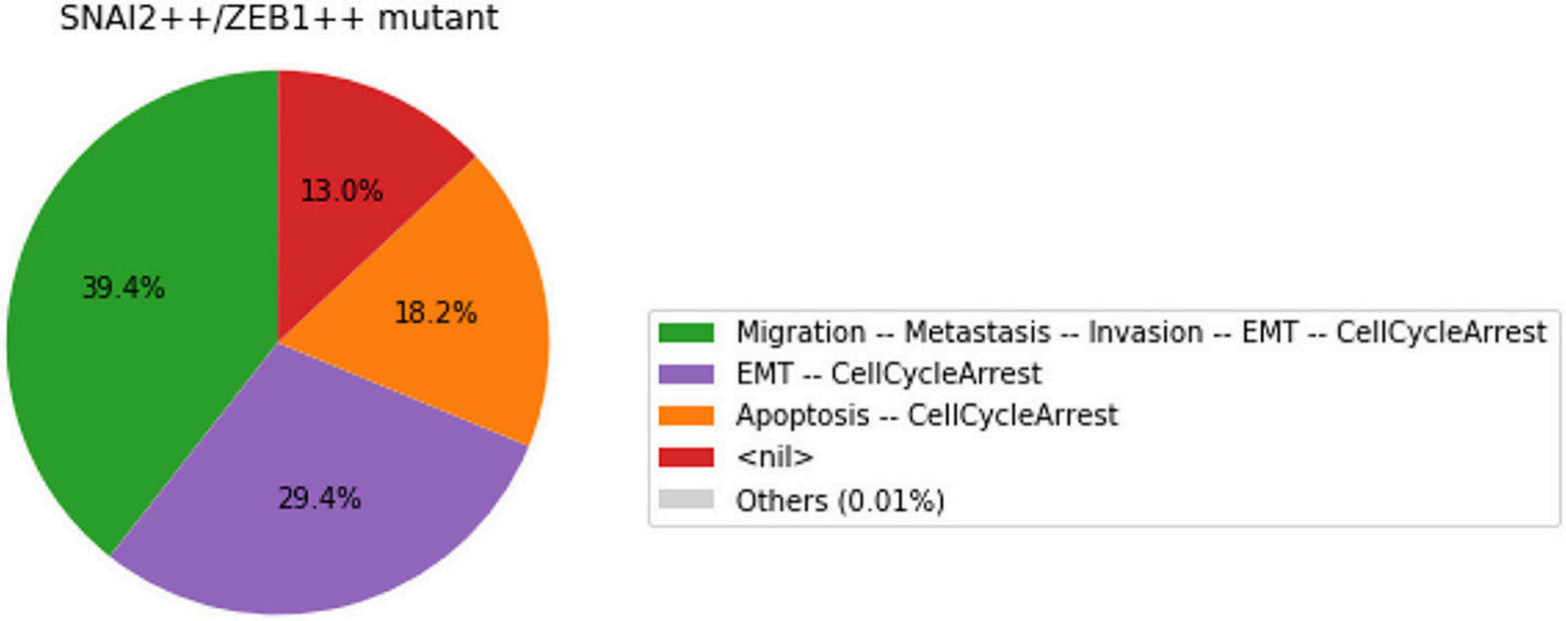
Graphical output resulting from the input code: In [29].

**Figure 12:**
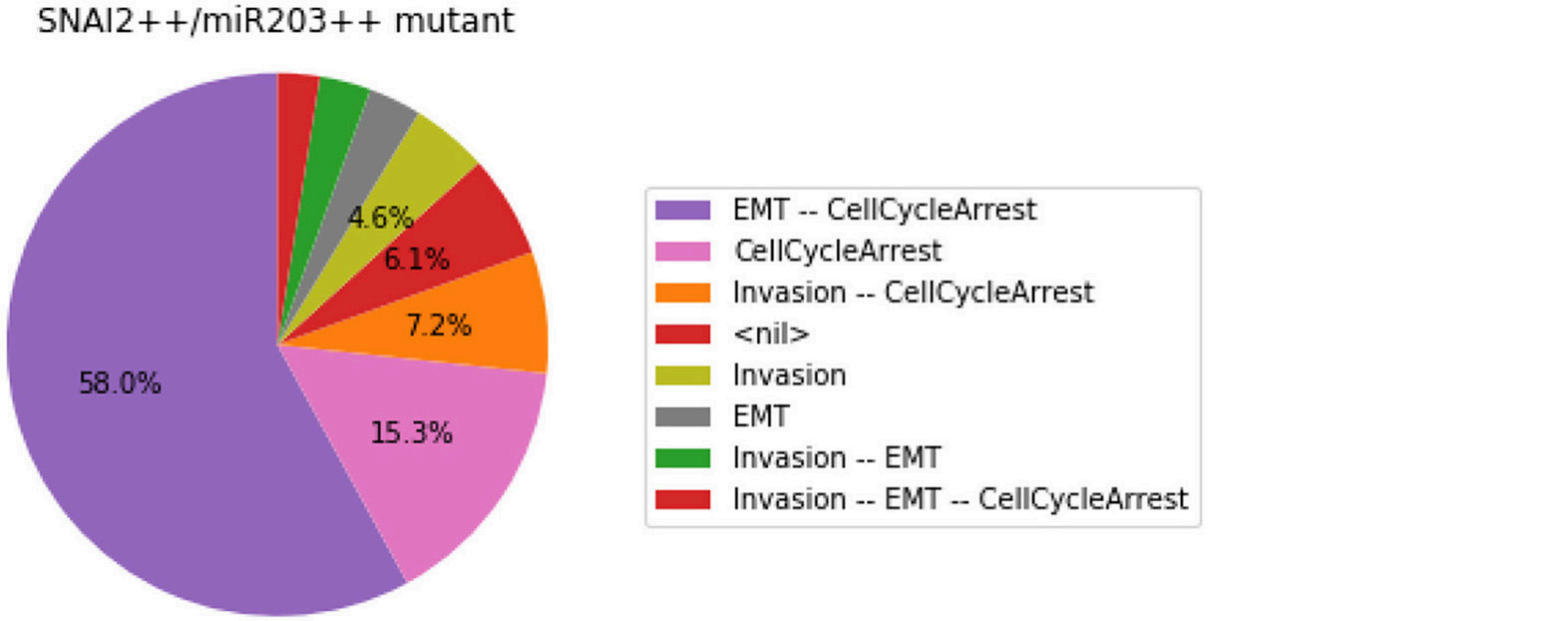
Graphical output resulting from the input code: In [29].

**Figure 13:**
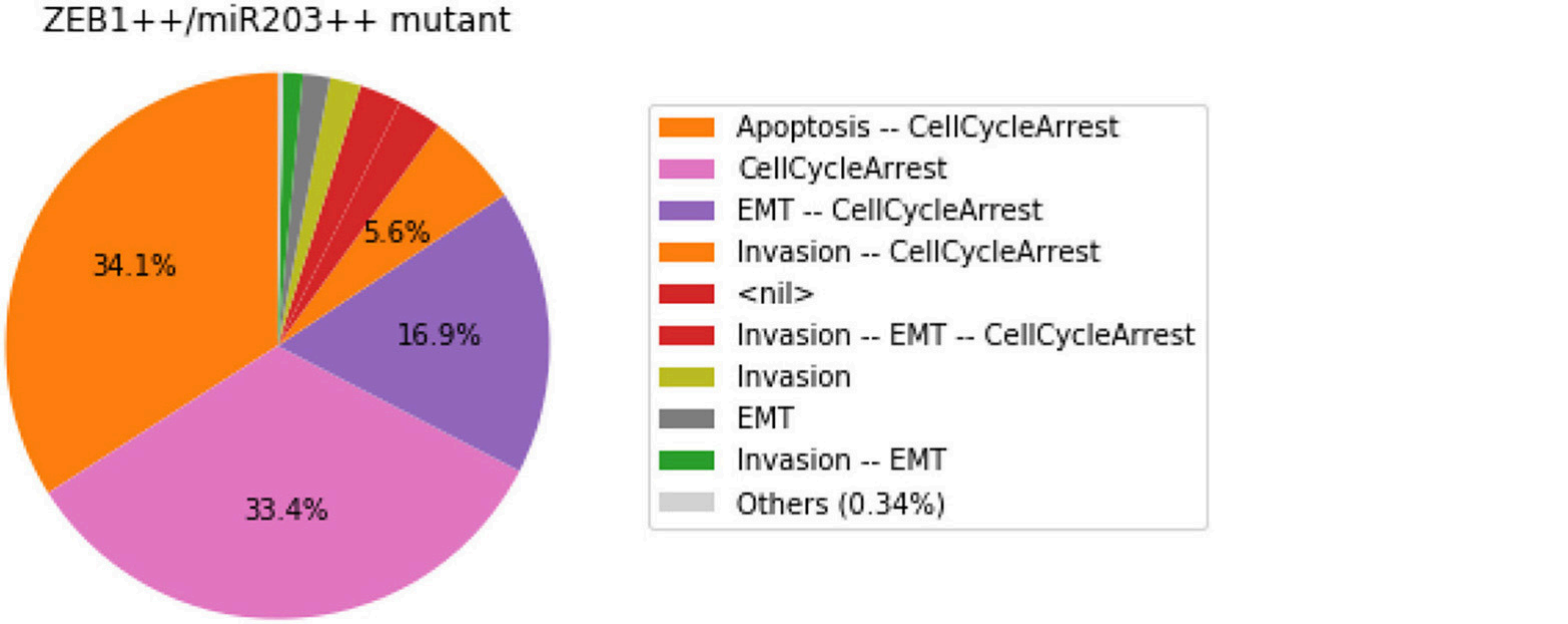
Graphical output resulting from the input code: In [29].

The resulting graphics are reproduced in Figures 8–13.

Note that only one of the pie charts shows an absence of apoptotic state: the SNAI2++/miR203++ double mutant (Figure 13).

This can be formally verified with NuSMV, as we did for the Notch++/p53-- mutant:


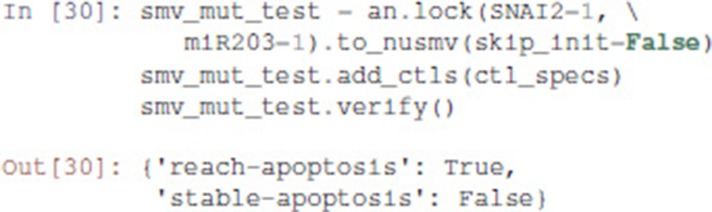


## 4. Anticipated results

With this protocol, we showed how the Python interface and Jupyter integration of GINsim, bioLQM, MaBoSS, and Pint ease the delineation of sophisticated re-executable computational analyses of qualitative models of biological networks, combining and chaining different software with a unified interface.

Leaning on the CoLoMoTo Docker image and on the companion Jupyter notebook, we have demonstrated the benefits of this framework by revisiting the analysis of a recent Boolean model of the signaling network controlling cancer cell metastasis. We could reproduce results previously obtained with GINsim and MaBoSS, which demonstrate that the Notch++/p53-- double mutant can suppress the apoptotic outcome. Furthermore, a formal analysis of trajectories with Pint enabled us to deduce novel “anti-apoptotic” combinations of mutations, including a triple mutant that forbids even transient activation of apoptosis, which were subsequently quantified using MaBoSS.

The predicted of mutations point to potential synergistic genetic interactions underlying uncontrolled tumor proliferation. These combinations would deserve further analysis, in particular regarding potential correlations with specific clinical outcomes. For example, one could check whether the loss of apoptosis triggering correlates with higher tumor grades.

Similar computational analyses could be performed to predict combinations of perturbations enforcing the existence of a given stable phenotype, e.g., apoptosis, which could then serve as a basis to design novel therapeutic strategies.

## Author contributions

NL, AN, CH, LP implemented the necessary Python modules, their integration in the Jupyter interface, and the Docker image. NL, AN, GS, DT, AZ, LC, LP participated to the general design of the notebook. All authors participated to the writing of the article.

### Conflict of interest statement

The authors declare that the research was conducted in the absence of any commercial or financial relationships that could be construed as a potential conflict of interest.

## Footnotes

^1^Available at http://colomoto.org/notebook

^2^https://docker.com

^3^https://jupyter.org

^4^You may have to use pip3 instead of pip depending on your configuration.

## Supplementary material

The supplemental data “Notebook” (Data Sheet 1) includes the source notebook file (.ipynb extension) which can be uploaded and executed within the Jupyter interface of the CoLoMoTo notebook, using the Docker image colomoto/colomoto-docker:2018-05-29. We further provide a static HTML file to preview the Jupyter rendering of the notebook, along with the file containing the Boolean model used.

The supplemental data “Fixpoints” (Data Sheet 2) gives the complete description of the fixpoints computed by code cell In [6].
