# Supplementary material for: Prediction of Mutations to Control Pathways Enabling Tumor Cell Invasion with the CoLoMoTo Interactive Notebook (Tutorial)
Source: Front Physiol. 2018 Jul 6;9:787. doi: 10.3389/fphys.2018.00787 (PMC6043725; doi:10.3389/fphys.2018.00787)
Supplement: Supplementary file 1 [file Data_Sheet_1.ZIP › notebook.html]

Usecase - Mutations enabling tumour invasion


# Prediction of Mutations to Control Pathways Enabling Tumour Cell Invasion with the CoLoMoTo Interactive Notebook (Tutorial)¶

**Authors**: Nicolas Levy1,2, Aurélien Naldi3, Céline Hernandez3, Gautier Stoll4-8, Denis Thieffry3, Andrei Zinovyev9-11, Laurence Calzone9-11, Loïc Paulevé1,\*

> 1 LRI UMR 8623, Université Paris-Sud, CNRS, Université Paris-Saclay, Orsay, France;
> 2 École Normale Supérieure de Lyon, France;
> 3 Computational Systems Biology team, Institut de Biologie de l’Ecole Normale Supérieure, CNRS UMR8197, INSERM U1024, École Normale Supérieure, PSL Université, Paris, France;
> 4 Université Paris Descartes/Paris V, Sorbonne Paris Cité, Paris, France;
> 5 Équipe 11 labellisée Ligue Nationale contre le Cancer, Centre de Recherche des Cordeliers; Paris, France;
> 6 Institut National de la Santé et de la Recherche Médicale, U1138; Paris, France;
> 7 Université Pierre et Marie Curie, Paris, France;
> 8 Metabolomics and Cell Biology Platforms, Gustave Roussy Cancer Campus; Villejuif, France;
> 9 Institut Curie, PSL Research University, Paris, France;
> 10 INSERM, U900, Paris, France;
> 11 MINES ParisTech, PSL Research University, CBIO-Centre for Computational Biology, Paris, France

### Abstract¶

> Boolean and multi-valued logical formalisms are increasingly used to model complex cellular networks. To ease the development and analysis of logical models, a series of software tools have been proposed, often with specific assets.
> However, combining these tools typically implies a series of cumbersome software installation and model conversion steps. In this respect, the *CoLoMoTo Interactive Notebook* provides a joint distribution of several logical modelling software tools, along with an interactive web Python interface easing the chaining of complementary analyses.
>
> Our computational workflow combines (1) the importation of a GINsim model and its display, (2) its format conversion using the Java library BioLQM, (3) the formal prediction of mutations using the OCaml software Pint, (4) the model checking using the C++ software NuSMV, (5) quantitative stochastic simulations using the C++ software MaBoSS, and (6) the visualisation of results using the Python library matplotlib.
> We rely on the CoLoMoTo Interactive Notebook to provide a thoroughly documented and executable notebook of our computational analysis.
>
> To illustrate our approach, we use a recent Boolean model of the signalling network controlling tumour cell invasion and migration. Our model analysis culminates with the prediction of sets of mutations presumably involved in a metastatic phenotype.
> Beyond the specific application considered here, the resulting Jupyter notebook constitutes a tutorial for the use and combination of several logical modelling tools, which can be easily adapted to analyse other models. Furthermore, the notebook web interface and the companion Docker distribution of logical modelling tools greatly facilitate the production of accessible and reproducible model analysis workflows.
>
> **Keywords**: Boolean networks, stochastic simulations, model verification, software tools, reproducibility

# Introduction¶

Boolean and multi-valued logical formalisms are increasingly used to model complex cellular networks (see e.g. Helikar et al. (2012);
Zañudo et al. (2015);
Collombet et al. (2017)). A logical model is usually defined in three steps:

1. The delineation of a regulatory graph, where the vertices (nodes) represent signalling or regulatory components (proteins, genes, microRNAs, etc.), while the arcs (arrows) represent regulatory interactions between pairs of components. These arcs are labelled by a sign: positive in the case of activation, negative in the case of an inhibition (multiple arcs between two nodes may be considered but are not used here).
2. A discrete variable is associated with each node. In the simplest cases, as hereafter, these variables are Boolean, *i.e.* they can take only two values (0 or 1), denoting the absence/inactivity or the presence/activity of the corresponding components.
3. Finally, a logical rule is associated with each component to specify the combinations enabling its activation. More precisely, this rule combines the different variables corresponding to the regulatory components using the logical negation (denoted by `!`), conjunction (denoted by `&`) and disjunction (denoted by `|`). For example, the rule associated with the component GF in the model considered below is `!CDH1 & (GF | CDH2)`, which reads as "the component GF will be activated in the absence of CDH1 and in the presence of CDH2 or GF itself". In other words, CDH2 is required transiently for GF activation, in the absence of CDH1.

To support the development and analysis of logical models, a series of software tools have been proposed, often with specific assets (Naldi et al., 2009;
Klarner et al., 2017;
Paulevé, 2017;
Stoll et al., 2017).

The *CoLoMoTo Interactive Notebook*1 (Naldi et al., 2018b) relies on Docker^[https://docker.com] and Jupyter^[https://jupyter.org] technologies to assist on editing and sharing reproducible analysis workflows for logical models.
In addition to the distribution of a set of software tools to define and analyse Boolean and multi-valued networks, a unified Python interface for each of the integrated tools is provided, greatly easing the execution and chaining of complementary analyses.

This protocol describes in details the usage of the CoLoMoTo Interactive Notebook to provide a reproducible analysis of a recently published model of the signalling network controlling tumour cell invasion and migration. More specifically, we combine different tools (Table 1) to compute the model stable states, perform stochastic simulations, compute (sets of) mutations controlling the reachability of specific stable states, and evaluate their efficiency.

**Table 1** List of software tools used in this notebook

| Tool | Website | Role in this notebook |
| --- | --- | --- |
| GINsim | ginsim.org | Model input and display, conversion to bioLQM and NuSMV |
| bioLQM | colomoto.org/biolqm | Fixpoint computation, conversion to MaBoSS and Pint |
| MaBoSS | maboss.curie.fr | Stochastic simulations, assess impact of mutations on propensity of reaching phenotypes |
| Pint | loicpauleve.name/pint | Formal prediction of mutants |
| NuSMV | nusmv.fbk.eu | Formal verification of phenotypes reachability and stability |

# Materials and Equipment¶

## Executable and reproducible model analysis¶

This protocol has been actually edited entirely as a Jupyter notebook before being converted to a LaTeX document for journal-specific editing purposes. The original notebook file is provided as supplemental material. It can also be visualised and downloaded for execution in the CoLoMoTo Interactive Notebook at https://nbviewer.jupyter.org/gist/pauleve/a86717b0ae8750440dd589f778db428f/Usecase - Mutations enabling tumour invasion.ipynb.

The blocks beginning with `In [..]` correspond to Jupyter *code cells*, which contain the Python instructions to execute. When relevant, the blocks beginning with `Out [..]` display the result of the last instruction of the corresponding code cell.

Provided Docker and Python are installed, the CoLoMoTo Interactive notebook can be installed by typing and executing the following command2 on GNU/Linux, macOS, and Microsoft Windows:

```
pip install -U colomoto-docker
```

Once installed, the notebook can be executed by typing

```
colomoto-docker -V 2018-05-29
```

The execution of this command will open a web page with the Jupyter notebook interface, enabling the loading and execution of the code.
Note that "SHIFT+ENTER" must be used to execute each code cell.
More information on `colomoto-docker` usage can be obtained by typing `colomoto-docker --help` and by visiting https://github.com/colomoto/colomoto-docker.

---

1. Available at https://github.com/colomoto/colomoto-docker↩
2. You may have to use `pip3` instead of `pip` depending on your configuration.↩

## Notebook preparation¶

This notebook makes use of the following Python modules:

In [1]:

```
import ginsim
import biolqm
import maboss
import pypint
from colomoto_jupyter import tabulate # for fixpoint table display
from itertools import combinations  # for iterating over sets
import matplotlib.pyplot as plt # for modifying plots
```

This notebook has been executed using the docker image `colomoto/colomoto-docker:2018-05-29`

You are using Pint version 2018-03-22 and pypint 1.4.1

# Stepwise Procedures¶

## Model¶

We analyse a Boolean model of the signaling network controlling cell tumour invasion, which was recently reported in Cohen et al. (2015).
This model can be loaded directly from the GINsim model repository at http://ginsim.org/models\_repository.

We first show how to use GINsim (Naldi et al., 2018a) to fetch and parse the GINML file (GINsim graph-based XML format, encapsulated in a zginml archive) and display the regulatory graph of the network.
To load the model, we copied the URL of the `.zginml` file from the model repository page at http://ginsim.org/node/191.
The file is also available as supplemental data (suppMat\_Model\_Master\_Model.zginml).

In [2]:

```
lrg = ginsim.load("http://ginsim.org/sites/default/files/SuppMat_Model_Master_Model.zginml")
```

Downloading 'http://ginsim.org/sites/default/files/SuppMat\_Model\_Master\_Model.zginml'

The regulatory graph (using the graphical setting specified in the model file) can be displayed with the following command:

In [3]:

```
ginsim.show(lrg)
```

Out[3]:

In this regulatory graph, the grey boxes denote input and output vertices (nodes). Green arrows and red T arrows respectively denote activatory and inhibitory interactions. A set of rules combining the vertices with the Boolean operators NOT, AND, and OR, which must be consistent with the regulatory graph, then allows the computation of enabled transitions for each network state. These rules have been defined in Cohen et al. (2015) and are specified within the GINsim model.

## Identification of stable states¶

First, we compute the complete list of logical stable states (or fixpoints) of the model using the Java library `bioLQM` (Naldi, submitted).
We thus need to convert the GINsim model into bioLQM:

In [4]:

```
lqm = ginsim.to_biolqm(lrg)
```

At that stage, `lrg` is a Python object representing the model suitable for GINsim, and `lqm` is a Python object representing the equivalent model suitable for bioLQM.

The list of stable states of a bioLQM model is computed as follows:

In [5]:

```
fixpoints = biolqm.fixpoints(lqm)
```

Here, `fixpoints` is a Python list of states. A state is encoded as a Python association table (dictionary), which maps each node of the network to a value.

For a nice display of the list of stable states, one can use the `tabulate` function provided in the `colomoto_jupyter` Python library, imported at the beginning of the notebook:

In [6]:

```
tabulate(fixpoints)
```

Out[6]:

|  | ECMicroenv | DNAdamage | Metastasis | Migration | Invasion | EMT | Apoptosis | CellCycleArrest | GF | TGFbeta | p21 | CDH1 | CDH2 | VIM | TWIST1 | SNAI1 | SNAI2 | ZEB1 | ZEB2 | AKT1 | DKK1 | CTNNB1 | NICD | p63 | p53 | p73 | miR200 | miR203 | miR34 | AKT2 | ERK | SMAD |
| --- | --- | --- | --- | --- | --- | --- | --- | --- | --- | --- | --- | --- | --- | --- | --- | --- | --- | --- | --- | --- | --- | --- | --- | --- | --- | --- | --- | --- | --- | --- | --- | --- |
| 0 | 0 | 0 | 0 | 0 | 0 | 0 | 0 | 0 | 0 | 0 | 0 | 1 | 0 | 0 | 0 | 0 | 0 | 0 | 0 | 0 | 0 | 0 | 0 | 0 | 0 | 0 | 0 | 0 | 0 | 0 | 0 | 0 |
| 1 | 0 | 0 | 0 | 0 | 0 | 1 | 0 | 1 | 1 | 0 | 0 | 0 | 1 | 1 | 1 | 1 | 1 | 1 | 1 | 0 | 0 | 0 | 0 | 0 | 0 | 0 | 0 | 0 | 0 | 1 | 1 | 0 |
| 2 | 0 | 1 | 0 | 0 | 0 | 0 | 1 | 1 | 0 | 0 | 1 | 1 | 0 | 0 | 0 | 0 | 0 | 0 | 0 | 0 | 0 | 0 | 0 | 0 | 1 | 0 | 1 | 1 | 0 | 0 | 0 | 0 |
| 3 | 0 | 1 | 0 | 0 | 0 | 0 | 1 | 1 | 0 | 0 | 1 | 1 | 0 | 0 | 0 | 0 | 0 | 0 | 0 | 0 | 0 | 0 | 0 | 1 | 0 | 1 | 1 | 0 | 0 | 0 | 0 | 0 |
| 4 | 0 | 1 | 0 | 0 | 0 | 1 | 0 | 1 | 1 | 0 | 0 | 0 | 1 | 1 | 1 | 1 | 1 | 1 | 1 | 0 | 0 | 0 | 0 | 0 | 0 | 0 | 0 | 0 | 0 | 1 | 1 | 0 |
| 5 | 1 | 0 | 1 | 1 | 1 | 1 | 0 | 1 | 1 | 1 | 0 | 0 | 1 | 1 | 1 | 1 | 1 | 1 | 1 | 0 | 1 | 0 | 1 | 0 | 0 | 0 | 0 | 0 | 0 | 1 | 1 | 1 |
| 6 | 1 | 1 | 0 | 0 | 0 | 0 | 1 | 1 | 0 | 1 | 1 | 1 | 0 | 0 | 0 | 0 | 0 | 0 | 0 | 0 | 0 | 0 | 0 | 0 | 1 | 0 | 1 | 1 | 0 | 0 | 0 | 0 |
| 7 | 1 | 1 | 0 | 0 | 0 | 0 | 1 | 1 | 0 | 1 | 1 | 1 | 0 | 0 | 0 | 0 | 0 | 0 | 0 | 0 | 0 | 0 | 0 | 1 | 0 | 1 | 1 | 0 | 0 | 0 | 0 | 0 |
| 8 | 1 | 1 | 1 | 1 | 1 | 1 | 0 | 1 | 1 | 1 | 0 | 0 | 1 | 1 | 1 | 1 | 1 | 1 | 1 | 0 | 1 | 0 | 1 | 0 | 0 | 0 | 0 | 0 | 0 | 1 | 1 | 1 |

It results that the model has nine stable states, each corresponding to a row in the table, four of which enable apoptosis (rows with value 1 in fourth column "Apoptosis"). Note that the input node `DNAdamage` is also active in each of these four states.

A state can be visualised on the regulatory graph using GINsim. For example, the third stable state can be displayed using the following command:

In [7]:

```
ginsim.show(lrg, fixpoints[2])
```

Out[7]:

In this graph, the vertices shown in white or orange denote components that are OFF (value 0) or ON (value 1) respectively.

## Assessing the probabilities to reach alternative attractors using MaBoSS¶

MaBoSS (Stoll et al., 2017) is a C++ software enabling the stochastic simulation of Boolean networks by translating them into continuous time Markov processes. Each node activation and inactivation is associated with an *up* and a *down* *rate*, which specify the propensity of the corresponding transitions. From a given state, the simulation integrates all the possible node updates and derives a probability and a duration for each transition. By default, all transitions are assigned the same rate. For a given set of initial conditions, MaBoSS produces time trajectories and estimates probabilities of model states over the whole simulation time. Steady state distributions can thus be approximated, provided that a sufficient number of sufficiently long simulations have been performed.

The aim of this section is to reproduce part of the results obtained by Cohen et al. (2015), which show that a Notch (NICD) gain-of-function together with a p53 loss-of-function prevent reaching a stable apoptotic phenotype.

First, we convert the bioLQM model to MaBoSS:

In [8]:

```
wt_sim = biolqm.to_maboss(lqm)
```

The variable `wt_sim` is a Python object that gathers both the Boolean network rules and the settings for the simulations, including the transition rates.

### Simulation setup¶

The stochastic simulation of Boolean networks with MaBoSS requires the specification of several parameters.

#### Initial states¶

First, a distribution of initial states must be specified: each simulation then starts from a state sampled from this distribution. The distribution is determined by assigning a probability to start in state `0` or in state `1` to each node. By default, a node has a probability `1` to start in state `0`.

The `maboss` Python library provides *widgets* to ease the assignment of this initial distribution.
The following code enables the definition of a distribution of initial states with all nodes at `0`, but `DNAdamage` and `ECMicroenv` with equiprobable `0` and `1` values.
After pressing "OK", the notebook cell will be replaced by the actual Python call resulting in equal probabilities for these two nodes to start in active or inactive states.

In [9]:

```
maboss.wg_set_istate(wt_sim)
```

In [9]:

```
#maboss.wg_set_istate(wt_sim)
maboss.set_nodes_istate(wt_sim, ["DNAdamage", "ECMicroenv"], 
                          [0.5, 0.5])
```

#### Output nodes¶

Using MaBoSS, we can focus on the *output* nodes and ignore the other nodes, which enable us to identify the corresponding phenotypes. This can be done using the following code:

In [10]:

```
#maboss.wg_set_output(wt_sim)
wt_sim.network.set_output(('Metastasis', 'Migration', 'Invasion', 
                           'EMT', 'Apoptosis', 'CellCycleArrest'))
```

#### Simulation parameters¶

The `update_parameters` method can be used to specify several parameters for the stochastic simulation algorithm. We show below the complete list of parameters with the values obtained by default when translating a model from GINsim.
The method can be called with any subset of these parameters.

Among the parameter list, `sample_count` corresponds to the number of simulations performed to compute statistics, while `max_time` is the maximum (simulated) duration of a trajectory. Note that for a proper estimation of probabilities of the stable states, `max_time` needs to be long enough for the simulation to reach an asymptotic solution.

In [11]:

```
wt_sim.update_parameters(discrete_time=0, use_physrandgen=0,
        seed_pseudorandom=100, sample_count=50000,
        max_time=75, time_tick=0.1, thread_count=4,
        statdist_traj_count=100, statdist_cluster_threshold=0.9)
```

### Simulation of the wild-type model¶

The object `wt_sim` represents the input of MaBoSS, encompassing both the network and simulation parameters.
The simulations are triggered with the `.run()` method and return a Python object for accessing the results.

In [12]:

```
%time wt_results = wt_sim.run()
```

```
CPU times: user 0 ns, sys: 5.24 ms, total: 5.24 ms
Wall time: 3.26 s
```

The resulting object gives access to the output data generated by MaBoSS.
It includes notably the mean probability over time for the activity of the output states integrated over all the performed simulations.

The function `plot_piechart` displays proportionally the mean probability of each output state at the *last* time point.
Provided the simulation time has been set high enough, this gives an approximation of the probabilities of the stable states reachable from the specified initial conditions.

In [13]:

```
wt_results.plot_piechart()
```

In this chart, a state is described by the set of its active output nodes and is associated to a phenotype.
For instance, the "`<nil>`" phenotype has all output nodes set to 0, which was referred to as the "homeostatic state" in the original article;
in the case of the "`Apoptosis -- CellCycleArrest`" phenotype, the two output nodes `Apoptosis` and `CellCycleArrest` are simultaneously active, while the other output nodes are inactive;
the "`EMT -- CellCycleArrest`" phenotype denotes cells that have gone through the epithelial to mesenchymal transition, but did not invade the tissue, hence the output nodes `Invasion`, `Migration` and `Metastasis` are inactive;
finally the "`Migration -- Metastasis -- Invasion -- EMT -- CellCycleArrest`" phenotype corresponds to a metastatic state, *i.e.* to cells that went through EMT, invaded the tissue and migrated to a distant site.

From this plot, we can deduce that, from the specified set of initial conditions, the apoptotic state (orange section), the EMT (purple section) and the metastatic states (green section) can be reached (the proportion of simulations that reached none of these phenotypes correspond to the red section).

The mean value of each output node during the simulations can be plotted with the following command:

In [14]:

```
wt_results.plot_node_trajectory(until=40)
```

### Simulation of double mutant Notch++/p53--¶

In the original article (Cohen et al., 2015), the authors analysed the double Notch++/p53-- mutant, *i.e.*, the combination of a Notch gain-of-function combined with a p53 loss-of-function, showing that all trajectories lead to a metastatic state.

A mutant can be configured by copying the wild-type model, and use the `mutate` method to model the desired gains and losses of function:

In [15]:

```
mut_sim = wt_sim.copy()
mut_sim.mutate("p53", "OFF")
mut_sim.mutate("NICD", "ON")
```

The modified model can then be simulated exactly as for the wild-type case:

In [16]:

```
%time mut_results = mut_sim.run()
```

```
CPU times: user 3.97 ms, sys: 971 µs, total: 4.94 ms
Wall time: 3.24 s
```

In [17]:

```
mut_results.plot_piechart()
```

Using the same parameters as for the wild-type model, all the trajectories obtained for the double mutant model reach the metastatic invasive state exclusively. This suggests that such a double mutation can be responsible for a loss of apoptotic capability of cancer cells.

## Formal analysis with Pint and NuSMV¶

In the above section, the conclusion regarding the loss of apoptotic stable state relies on stochastic simulations, which, in general, may not offer a complete coverage of the possible trajectories. Therefore, one may want to formally verify whether the loss of reachable stable apoptosis state is total or not.
First, we show how to use Pint (Paulevé, 2017) to predict combinations of mutations which are guaranteed to prevent the activation of apoptosis.
Next, we use the software NuSMV (Cimatti et al., 2002) to evaluate formally the Notch++/p53-- double mutant.
Finally, we use MaBoSS to assess the efficiency of new combinations of mutations predicted by Pint.

### Formal predictions of mutations from the wild-type model¶

Pint implements formal methods that allow deducing combinations of mutations guaranteed to block the reachability of a given state.

First, we convert the bioLQM model to Pint:

In [18]:

```
an = biolqm.to_pint(lqm)
```

Then, we transfer the initial conditions defined in MaBoSS to the Pint model `an`. Like MaBoSS, Pint supports multiple initial values for a single node. However, in contrast to MaBoSS, Pint does not consider probability distributions.

In [19]:

```
an.initial_state.update(wt_sim.get_initial_state())
an.initial_state.changes()   # display non-default (0) initial value
```

Out[19]:

```
{'ECMicroenv': (0, 1), 'DNAdamage': (0, 1)}
```

Given a (partial) state specification, Pint provides the method `oneshot_mutations_for_cut`, which returns different sets of mutations guaranteed to prevent any trajectory from any possible initial state to reach, *even transiently*, the specified state.

In [20]:

```
%time an.oneshot_mutations_for_cut(Apoptosis=1, \
                exclude={"ECMicroenv", "DNAdamage"})
```

This computation is an *under-approximation*: returned mutations are all valid, but they may be non-minimal, and some solutions may be missed.

Limiting solutions to mutations of at most 5 automata. Use `maxsize` argument to change.

```
CPU times: user 5.2 ms, sys: 1.52 ms, total: 6.73 ms
Wall time: 158 ms
```

Out[20]:

```
[{'ZEB2': 1},
 {'AKT1': 1},
 {'AKT2': 1},
 {'ERK': 1},
 {'SNAI2': 1, 'ZEB1': 1, 'NICD': 1},
 {'SNAI2': 1, 'ZEB1': 1, 'p63': 0},
 {'SNAI2': 1, 'ZEB1': 1, 'miR203': 1},
 {'SNAI2': 1, 'NICD': 1, 'p73': 0},
 {'SNAI2': 1, 'p63': 0, 'p73': 0},
 {'SNAI2': 1, 'p73': 0, 'miR203': 1},
 {'ZEB1': 1, 'NICD': 1, 'p53': 0},
 {'ZEB1': 1, 'p63': 0, 'p53': 0},
 {'ZEB1': 1, 'p53': 0, 'miR203': 1},
 {'NICD': 1, 'p53': 0, 'p73': 0},
 {'p63': 0, 'p53': 0, 'p73': 0},
 {'p53': 0, 'p73': 0, 'miR203': 1}]
```

Among the returned mutation sets, one can spot the mutation `{'NICD': 1, 'p53': 0, 'p73': 0}`, which combines a gain-of-function of Notch (`'NICD': 1`) with a loss-of-function of p53 (`'p53': 0`), along with a loss-of-function of p73 (`'p73': 0`).

Noteworthy, forbidding *transient* reachability entails a stronger constraint than just preventing any *stable* state with the specified property. Indeed, some mutations may remove the stability of the specified states, while some trajectories may still traverse these states, but only transiently.

Therefore, the sets of mutations returned by Pint, albeit correct, might be non-minimal for controlling only the long-term dynamics of the system. Finally, note that the analysis of Pint can give incomplete results. This is due to the technology on which the computation relies (static analysis), which allows addressing very large scale networks.

### Revisiting the Notch++/p53-- double mutant¶

We will first formally analyse the Notch++/p53-- double mutant to show that asymptotic apoptosis is forbidden, although transient activation of apoptosis node might still be possible.

One can apply a mutation on a Pint model using the `lock` method. A new model is returned with a constant value for the corresponding nodes.

In [21]:

```
mut_an = an.lock(NICD=1, p53=0)
```

Then, we use the temporal logic CTL (Clarke et al., 1981) to specify formally the dynamical properties to verify.
CTL expression can be built using the `colomoto.temporal_logics` Python module.

In [22]:

```
from colomoto.temporal_logics import *
```

First, the existence of a trajectory leading to a *transient* state where `Apoptosis` is active can be specified as follows:

In [23]:

```
transient_apoptosis = EF(S(Apoptosis=1))
```

`EF` is a temporal logic operator that is true if there exists at least one trajectory leading to a state verifying the properties given as argument. Here the property `S(Apoptosis=1)` specifies that the state has the node `Apoptosis` active.

Next, the existence of a trajectory leading to a *stable* `Apoptosis` activation can be specified as follows:

In [24]:

```
stable_apoptosis = EF(AG(S(Apoptosis=1)))
```

Here, `AG` enforces that *all* the states reachable via any trajectory have the node `Apoptosis` active.

Finally, we gather these two properties in a Python dictionary for later use:

In [25]:

```
ctl_specs = {
    "reach-apoptosis": transient_apoptosis,
    "stable-apoptosis": stable_apoptosis
}
```

The adequation of a model with a CTL property can be assessed using a *model-checker* such as NuSMV (Abou-Jaoudé et al., 2015).

Pint provides a conversion to NuSMV models. By default, the NuSMV model considers any initial state. With the `skip_init=False` option, we enforce that the properties are verified only from the initial states defined earlier.

In [26]:

```
smv = mut_an.to_nusmv(skip_init=False)
```

We then add the properties defined above, and ask NuSMV to verify them.

In [27]:

```
smv.add_ctls(ctl_specs)
%time smv.verify()
```

```
CPU times: user 4.66 ms, sys: 0 ns, total: 4.66 ms
Wall time: 12.3 s
```

Out[27]:

```
{'reach-apoptosis': True, 'stable-apoptosis': False}
```

Interestingly, the Notch++/p53-- double mutant can still reach an apoptotic state, but only transiently: the property `stable-apoptosis` being false, it is guaranteed that all trajectories eventually lead to stable apoptosis inactivation.

To complete our analysis, we now consider the triple mutant obtained by adding a loss-of-function of `p73`. As predicted by Pint, transient reachability of apoptosis is impossible in this triple mutant. We can use NuSMV to further verify that it is the case, using the following code:

In [28]:

```
smv_mut3 = an.lock(NICD=1, p53=0, p73=0).to_nusmv(skip_init=False)
smv_mut3.add_ctls(ctl_specs)
smv_mut3.verify()
```

Out[28]:

```
{'reach-apoptosis': False, 'stable-apoptosis': False}
```

### Analysis of formally predicted SNAI2++/ZEB1++/miR203++ triple mutant¶

The mutant combinations predicted with Pint should be refined when the aim is to control specifically stable behaviours. In general, given a set of mutations guaranteed to block any transient activation of a node, one may verify whether only a subset of them are sufficient to achieve proper control of the sole stable states.

We show here how we can take advantage of the Python environment to provide a small program, which, for each subset of mutations of a multiple mutant (here a triple gain-of-function for SNAI2, ZEB1 and miR203), performs stochastic simulations with MaBoSS to assess the probabilities to reach the different stable behaviours from the specified set of states.

The computation can take a couple of minutes. The results are shown in a graphical form (coloured pie charts) for each single and double loss-of-function combination. In the pie charts, "Others" regroup states with an individual probability less than 1%, which often correspond to simulated trajectories having not reached an attractor in the given amount of time.

In [29]:

```
formal_mutant = {'SNAI2': 1, 'ZEB1': 1, 'miR203': 1}
for i in [1, 2]:
    # for any subset of mutations of size 1 then 2
    for mutants in combinations(formal_mutant, i):
        # copy the wild-type MaBoSS model
        masim = wt_sim.copy()
        # apply the mutations
        for m in mutants:
            masim.mutate(m, "ON" if formal_mutant[m] else "OFF")
        # run the simulations
        mares = masim.run()
        # plot the piechart of stable states
        mares.plot_piechart()
        # print the mutation in the title
        def mutname(m):
            return m + ("++" if formal_mutant[m] else "--")
        name = "/".join(map(mutname, mutants))
        plt.title("%s mutant" % name)
```

Note that only one of the pie charts shows an absence of apoptotic state: the SNAI2++/miR203++ double mutant.

This can be formally verified with NuSMV, as we did for the Notch++/p53-- mutant:

In [30]:

```
smv_mut_test = an.lock(SNAI2=1, miR203=1).to_nusmv(skip_init=False)
smv_mut_test.add_ctls(ctl_specs)
smv_mut_test.verify()
```

Out[30]:

```
{'reach-apoptosis': True, 'stable-apoptosis': False}
```

# Anticipated Results¶

With this protocol, we showed how the Python interface and Jupyter integration of GINsim, bioLQM, MaBoSS, and Pint ease the delineation of sophisticated re-executable computational analyses of qualitative models of biological networks, combining and chaining different software with a unified interface.

Leaning on the CoLoMoTo Docker image and on the companion Jupyter notebook, we have demonstrated the benefits of this framework by revisiting the analysis of a recent Boolean model of the signalling network controlling cancer cell metastasis.
We could reproduce results previously obtained with GINsim and MaBoSS, which demonstrate that the Notch++/p53-- double mutant can suppress the apoptotic outcome.
Furthermore, a formal analysis of trajectories with Pint enabled us to deduce novel "anti-apoptotic" combinations of mutations, including a triple mutant that forbids even transient activation of apoptosis, which were subsequently quantified using MaBoSS.

The resulting combinations of mutations point to potential synergistic genetic interactions underlying uncontrolled tumour proliferation.
These combinations would deserve further analysis, in particular regarding potential correlations with specific clinical outcomes.
For example, one could check whether the loss of apoptosis triggering correlates with higher tumour grades.

Similar computational analyses could be performed to predict combinations of perturbations enforcing the existence of a given stable phenotype, *e.g.* apoptosis, which could then serve as a basis to design novel therapeutic strategies.

# Conflict of Interest Statement¶

The authors declare that the research was conducted in the absence of any commercial or financial relationships that could be construed as a potential conflict of interest.

# Author Contributions¶

NL, AN, CH, LP implemented the necessary Python modules, their integration
in the Jupyter interface, and the Docker image.
NL, AN, GS, DT, AZ, LC, LP participated to the general design of the notebook.
All authors participated to the writing of the article.

# Funding¶

DT and CH acknowledge support from the French Plan Cancer, in the context of the projects CoMET (2014-2017) and SYSTAIM (2015-2019).
DT and AN acknowledge support from the French Agence Nationale pour la
Recherche (ANR), in the context of the project SCAPIN [ANR-15-CE15-0006-01].
AZ and LC acknowledge support from ITMO Cancer, in the context of the INVADE grant (Call Systems Biology 2012), and
from the EU ERACoSysMed programme, in the context of the COLOSYS project.
AZ, LC, and LP acknowledge support from the ANR in context of the ANR-FNR project AlgoReCell [ANR-16-CE12-0034].
LP acknowledge support from Paris Ile-de-France Region (DIM RFSI) and
Labex DigiCosme [ANR-11-LABEX-0045-DIGICOSME] operated by ANR as part of the program
"Investissement d'Avenir" Idex Paris-Saclay [ANR-11-IDEX-0003-02].

# Bibliography¶

Abou-Jaoudé, W., Monteiro, P. T., Naldi, A., Grandclaudon, M., Soumelis, V., Chaouiya, C., et al. (2015). Model checking to assess t-helper cell plasticity. *Front. Bioeng. Biotechnol.* 2, 86. doi:10.3389/fbioe.2014.00086.

Cimatti, A., Clarke, E., Giunchiglia, E., Giunchiglia, F., Pistore, M., Roveri, M., et al. (2002). “NuSMV 2: An opensource tool for symbolic model checking,” in *Computer aided verification* Lect. notes comput. sci., 241–268. doi:10.1007/3-540-45657-0\_29.

Clarke, E. M., and Emerson, E. A. (1981). Design and synthesis of synchronization skeletons using branching-time temporal logic. in *Logic of programs*, 52–71. doi:10.1007/BFb0025774.

Cohen, D. P. A., Martignetti, L., Robine, S., Barillot, E., Zinovyev, A., and Calzone, L. (2015). Mathematical modelling of molecular pathways enabling tumour cell invasion and migration. *PLoS Comput. Biol.* 11, e1004571. doi:10.1371/journal.pcbi.1004571.

Collombet, S., Oevelen, C. van, Sardina Ortega, J. L., Abou-Jaoudé, W., Di Stefano, B., Thomas-Chollier, M., et al. (2017). Logical modeling of lymphoid and myeloid cell specification and transdifferentiation. *Proc. Natl. Acad. Sci.* 114, 5792–5799. doi:10.1073/pnas.1610622114.

Helikar, T., Kowal, B., McClenathan, S., Bruckner, M., Rowley, T., Madrahimov, A., et al. (2012). The cell collective: Toward an open and collaborative approach to systems biology. *BMC Syst. Biol.* 6, 96. doi:10.1186/1752-0509-6-96.

Klarner, H., Streck, A., and Siebert, H. (2017). PyBoolNet: A python package for the generation, analysis and visualization of boolean networks. *Bioinformatics* 33, 770–772. doi:10.1093/bioinformatics/btw682.

Naldi, A. (2018). bioLQM: a java library for the manipulation and conversion of Logical Qualitative Models of biological networks. *bioRxiv*. doi:10.1101/287011.

Naldi, A., Berenguier, D., Fauré, A., Lopez, F., Thieffry, D., and Chaouiya, C. (2009). Logical modelling of regulatory networks with GINsim 2.3. *BioSystems* 97, 134–9. doi:10.1016/j.biosystems.2009.04.008.

Naldi, A., Hernandez, C., Abou-Jaoudé, W., Monteiro, P. T., Chaouiya, C., and Thieffry, D. (2018a). Logical modelling and analysis of cellular regulatory networks with GINsim 3.0. *Front. Physiol.* doi:10.3389/fphys.2018.00646.

Naldi, A., Hernandez, C., Levy, N., Stoll, G., Monteiro, P. T., Chaouiya, C., et al. (2018b). The CoLoMoTo Interactive Notebook: Accessible and Reproducible Computational Analyses for Qualitative Biological Networks. *Front. Physiol.* doi:10.3389/fphys.2018.00680.

Paulevé, L. (2017). Pint: a static analyzer for transient dynamics of qualitative networks with IPython interface. in *CMSB 2017 - 15th conference on Computational Methods for Systems Biology* Lect. notes comput. sci., 309–316. doi:10.1007/978-3-319-67471-1\_20.

Stoll, G., Caron, B., Viara, E., Dugourd, A., Zinovyev, A., Naldi, A., et al. (2017). MaBoSS 2.0: an environment for stochastic Boolean modeling. *Bioinformatics* 33, 2226–2228. doi:10.1093/bioinformatics/btx123.

Zañudo, R., Jorge G. T. AND Albert (2015). Cell fate reprogramming by control of intracellular network dynamics. *PLOS Comput. Biol.* 11, 1–24. doi:10.1371/journal.pcbi.1004193.
